# The Conical, Olive-Pointed, Gum Elastic Bougies in the Treatment of Organic Stricture

**Published:** 1874-03

**Authors:** John T. Wall

**Affiliations:** Tampa, Florida


### THE CONICAL, OLIVE-POINTED, GUM ELASTIC BOUGIES IN THE TREATMENT OF ORGANIC STRICTURE.

BY JOHN T. WALL, M. D., TAMPA, FLORIDA.

No subject in surgery has probably received more attention from the ablest minds in the profession than the treatment and cure of stricture. This is attested not only by the great amount of literature upon the subject, but also by the extraordinary number as well as variety of instruments which, from time to time, have been devised for its treatment.

Without entering into its pathology and maintaining it, with Sir Henry Thompson and others, to be a hypertrophy, the result of an inflammatory plastic exudation in and beneath the mucous membrane; or siding with M. Mercier in considering it an atrophy with tortuosity of the canal, or going into anything like a review of the subject of stricture, I shall merely give such facts and hints for its treatment, with French gum elastic bougies, as experience and observation have afforded me.

In all cases where gradual dilatation is the method of treatment adopted, these bougies are preferable for the following reasons: first, the absence of the risk of making false passages in inexpert hands; second, their introduction is not followed by any nervous or febrile disturbance; and then confinement to bed or room is not necessary, as, without detriment, the patient may pursue his usual avocations, provided they are not in themselves of a nature to produce or aggravate urethral affections. The only loss of time incurred will be in visiting the surgeon's office for a few minutes daily, or three or four times a week, for a month or so.

Even where the coarctation is so great as only to allow at first the passing of a filiform bougie, the treatment will soon demonstrate the feasibility of the oldest strictures being made to yield to this mild and almost painless method of dilatation. The flexibility of the more tenuous sizes allows them to follow the tortuosity of the canal with facility, by which violence to its salient points and angles is avoided, which violence is almost inevitable with a rigid instrument; the dictum of M. Mercier being here pertinent: "La sonde rigide ne cede pas, c'est l'obstacle qui cede devant elle." With them forcible or rapid dilation is not practicable, because the instrument bends or folds in the urethra if its size does not admit of its passing the stricture; consequently any force beyond firm but momentary pressure is neither requisite nor desirable. The following rules for their use comprise the main points to be observed. Of course, it is premised that the diagnosis of stricture has been made out:

It is well to commence with the smallest size, or, if necessary, filiform bougies, passing one after another—beginning with the smallest—until that size is reached which barely passes. This last should be allowed to remain in from two to five minutes, the length of time being determined, in each individual case, by the amount of irritability manifested on the part of the urethra. On the second, third or fourth day the operation should be repeated, the bougie last passed being first introduced and withdrawn, and the next in size introduced as far as it will go. After being in about a minute, a little firm pressure may again be applied, as by this time the contraction of the urethra, excited by the contact of the bougie, will, in all probability, have subsided. In like manner is each successive operation to be conducted, the last instrument passed being first introduced and withdrawn, and then the next larger size entered. The bougie being conical, though having a bulbous point, it usually requires several times' use of the same bougie before it passes; but it will be noticed at each successive operation that some advance is made. In all cases where the progress of the bougie may be interrupted by its point becoming engaged in mucous folds or lacunæ, it should be withdrawn a little and then pushed forward with a rotary movement. The same manœuvre is also often advantageously practiced to effect an entrance into the stricture, particularly where the plastic deposit assumes a crescentic character, encroaching on the bottom and sides of the canal, as is not unfrequently the case. If failure to introduce the smallest size, or filiform bougie, is experienced, a medium sized metallic catheter or sound may be passed down to the stricture, and firm but moderate pressure maintained for fifteen minutes, with a reasonable expectation of causing some slight opening of the orifice of the stricture, when the bougie may be again tried. Should the point of the filiform bougie become engaged in lacunæ, or rest, as it were, on the base of the coarctation, instead of withdrawing it, another and another should be introduced, until four or five may be thus lodged, an effort being made, of course, with each successive one to engage it in the orifice of the stricture. The object of this proceeding is to prevent the point of the last from occupying the place which has stopped the progress of the preceding bougie.

The bougies should be slightly warmed and well oiled. Injecting the urethra with sweet oil, as recommended by some of the English surgeons, possesses no doubt some advantages, and in difficult cases should be resorted to. By injecting with the oil, thorough lubrication of the canal down to the stricture at least is insured, even if penetration of the latter, either from the force used or by capillary attraction, is not effected. Again, when the bougie is merely oiled, its cone becomes denuded of the oil by the time it reaches the part to be acted upon, so that not only in the first endeavors to introduce bougies would the oil injection be beneficial, but at every subsequent operation throughout the treatment. The only possible objection to the practice is that of soiling the patient's linen; an objection truly insignificant except to the over fastidious in point of cleanliness.

After the larger sizes of the French bougies are reached, their lack of firmness renders them probably a little inferior to metallic instruments, but by removing their caps of wax and using stylets of lead, as suggested by Sir Henry Thompson, this inferiority is pretty effectually remedied. As good a position as any for the patient while introducing these flexible bougies is standing before the surgeon, a position by no means permissible in using metallic instruments.

In cases of long standing, where the bladder is in a condition bordering on, if not in an actual state of, chronic inflammation, the treatment will have to be conducted with extreme care. In such the intervals between operations will have to be longer than in ordinary cases, and the bougie allowed to remain but a very few minutes—say two or three. In all cases where much irritation follows the introduction of the bougie it is judicious to allow that to pretty well subside before again operating. This is particularly the case in such as have developed spasm with temporary retention, and a muco-purulent discharge. Here it may be necessary to order the warm hip-bath, administer alkalies, balsam copaiba, sandal wood oil or buchu, etc., and possibly apply leeches to the perineum. And of the use of leeches to the perineum it may not be amiss to state that, on the assertion of Mr. Trevan, their application often renders an apparently impassable stricture pervious to filiform bougies. He uses them in preference to chloroform for this purpose, and the practice is certainly more in consonance with common sense; for by this local depletion the tissues immediately surrounding the stricture are disgorged, and the latter rendered yielding, while but little if any such effect could be expected in organic stricture from the relaxation of chloroform.

After the calibre of the urethra has been restored to that of its meatus, the patient should be provided with one of the same kind of bougies of the requisite size, and instructed in the use of it on himself, as well as being warned of its probably becoming impaired with time and use, and consequently unsafe. The patient should also be instructed to introduce the bougie twice a week for a month or two; then once a week for several months, and finally at longer intervals for the remainder of his life. Both the surgeon and his patient should fully appreciate the dictum of Sir Henry Thompson, that "the treatment has yet to be devised which will remove absolutely and forever the occurrence of recontraction in a patient once the victim of organic stricture." From this it is reasonable to infer that the cure of stricture without the probability of its recurrence, if the occasional use of bougies is dispensed with, may be considered as not attainable with any of the several methods of treatment now in vogue. This fact, then, should incline the surgeon always to adopt that method which is the most simple and safe.

On this latter point, it may suffice to say that dilatation with metallic instruments, not unfrequently occasions severe constitutional disturbance with, possibly, inflammation of the whole urinary tract, to say nothing of the facility with which false passages may be produced with them. Treatment by divulsion has resulted in death sufficiently often to make any judicious surgeon hesitate in adopting this method, when regular dilatation can be practiced. The rapidity of the cure, which divulsion offers, can hardly counterbalance the hazard incurred of producing serious renal complications. Of urethrotomy and external division, it is only necessary to remind the young surgeon that they are grave operations, requiring, besides a practical hand, something more of an armamentarium than is usually found in the offices of the majority of general practitioners. Resort to these three last named methods can only be justifiable then, either where the more simple has failed, or where there are special indications forcing the surgeon to give speedy relief.

By a judicious and persevering trial with the French bougies, the surgeon will be equally surprised at, and gratified with, the ease with which old strictures of fifteen and twenty years' standing, are made to yield. My own success in thus treating old strictures has been so fortunate that now I am little disposed to resort to divulsion, or the other still more serious methods, in the management of this affection.

In forced catheterism, Bumstead's catheter, with filiform-bougie attachment is, in my opinion, superior to the tunnelled catheter, with whale-bone bougie of Prof. Gouley. Vollemine's rupture instrument, being also provided with a filiform bougie attachment, may be used in the same manner. In this procedure the filiform bougie is first passed and then attached to the point of either the catheter or rupture instrument, and being carried forward into the bladder, when it folds on itself, the possibility of making a false passage is pretty effectually obviated. The chief objection which may be urged against these instruments is, that suggested to me by Prof. Sam'l Logan, of New Orleans, viz: that there is some danger of the cap point being broken off within the urethra. While such an occurrence may be possible, I hardly think it likely to happen in careful hands. In the few instances in which I have used them I must acknowledge myself well pleased. However, it is not my intention to discuss the merits of these two ingenious methods of forcible catheterism, leaving it to time and experience to decide their respective claims to superiority and confidence.

In bringing this paper to a close, I would take this opportunity of stating that in three cases of incontinence of urine in girls of the respective ages of four, twelve and sixteen years, no benefit was derived from either belladonna, its alkaloid or chloral hydrate. The use of belladonna or its alkaloid, atropia, was persisted in until constitutional effects were manifested, as indicated by affecting sight with dilatation of the pupils. The chloral was given in gradually increasing doses at night until, in the cases of the two larger girls, the dose reached thirty grains. The two younger girls are sisters, and from what I can learn their infirmity is probably hereditary, their mother having been similarly affected until grown. These remedies, so much vaunted now and again in the various medical journals, may possibly prove successful in the case of males. In the latter sex, however, I have no experience to offer, as yet having had no occasion to try them.

